# Is the level of implementation linked with intervention outcomes? The process evaluation of the Veggie Van intervention to increase fruit and vegetable consumption in underserved communities

**DOI:** 10.21203/rs.3.rs-9889528/v1

**Published:** 2026-06-24

**Authors:** Christina Kasprzak, Rocco Paluch, Laurene Tumiel-Berhalter, Samina Raja, Lucia Lelone

**Affiliations:** University at Buffalo, State University of New York; University at Buffalo, State University of New York; University at Buffalo, State University of New York; University at Buffalo, State University of New York; University at Buffalo, State University of New York

**Keywords:** mobile market, implementation fidelity, community-based intervention, nutrition intervention, fruit and vegetable consumption, food security, implementation science

## Abstract

**Background:**

The Veggie Van (VV) model is a mobile produce market intervention previously shown to increase fruit and vegetable (F&V) consumption among lower-income individuals. Although the model was recently evaluated in a multi-state effectiveness trial, preliminary findings suggested challenges to implementing the model with fidelity. This study examined whether variation in implementation fidelity was associated with participant-level dietary, food security, and market utilization outcomes.

**Methods:**

This study involved a secondary analysis of participant outcome data from a longitudinal RCT conducted with 9 implementing organizations operating 17 mobile market sites (n=699 participants). Previously collected participant data included baseline and 12-month measures of F&V consumption from 24-hour dietary recalls and self-reported surveys, as well as food security assessed using the USDA 10-item Adult Food Security Survey Module. Participant outcome data were aggregated at the site level and linked with organizational implementation data collected throughout the intervention period. Implementing organizations completed monthly process measures surveys assessing adherence to core VV model components; responses were scored and aggregated into site-level implementation fidelity scores. Correlational analyses and generalized linear models examined associations between implementation fidelity and changes in F&V consumption, food security, and market utilization outcomes over 12 months.

**Results:**

The mean implementation fidelity score across sites was 40.6 out of 55 possible points. Higher overall fidelity was positively associated with greater increases in mean F&V consumption over 12 months (Pearson correlation coefficient [PCC]=0.56, p=0.02). Fidelity to the nutrition education component demonstrated a particularly strong association with change in F&V consumption (PCC=0.81, p<0.0001). These associations remained significant in regression analyses. In addition, sites operating markets for ≥10 months annually had 76% greater odds of participants shopping at the mobile market at least once during the intervention period (p=0.04). No significant associations were observed between implementation fidelity and changes in food security.

**Conclusions:**

Greater fidelity to the VV model, particularly implementation of nutrition education components, was associated with improved dietary outcomes in this secondary analysis of participant-level RCT data. Findings highlight the importance of evaluating implementation alongside effectiveness outcomes in community-based nutrition interventions and suggest that tailored implementation support strategies may strengthen delivery of intervention components most strongly associated with dietary change.

**Trial registration:**

The larger randomized controlled trial, the Veggie Van study, was registered at https://clinicaltrials.gov/ on January 29, 2020 (NCT04246593).

## BACKGROUND

The link between poor dietary quality, particularly lower dietary intake of fruits and vegetables (F&V), and risk of nutrition-related diseases is well-documented [1–3]. For example, the average American consumes about 2 ½ servings of F&V per day, which is well below the recommended daily intake of about 5 servings per day [4]. Poor diet quality is associated with an increased risk of cardiovascular diseases, metabolic disease, poor mental health, and certain types of cancer [5–11]. Further, food insecure individuals that lack access to affordable and nutrient-dense foods are more likely to have worse nutrition and a higher risk of diet-related diseases [12–15]. Lower-income and minoritized groups, such as Black and Hispanic households, constitute a disproportionate share of food insecure households in the US, further compounding disparities in economic and health outcomes compared with non-Hispanic White and higher-income populations [16–19].

Interventions that aim to influence dietary intake can be focused on the individual, but these downstream approaches fail to address other systemic issues and assume a consistent level of engagement from participants [20–22]. There is a need for multipronged interventions that address multiple dimensions of access to healthy food: acceptability, availability, affordability, accessibility, and accommodation [23–25]. Community-based food access strategies can serve as an impactful way to create sustained change in communities while supporting the localization of the food system and building capacity within communities [26–28]. One type of community-based strategy that has greatly increased in prevalence and popularity in the US is a mobile produce market, or mobile market [29–33]. Mobile markets are mobile healthy food retail markets, or “farmers markets on wheels,” that predominantly travel to communities experiencing limited food access, food insecurity, or health disparities [31–35]. Available research indicates that mobile markets can increase access to F&V and are perceived favorably among community members [29, 36–38]. Among the more rigorous experimental studies, mobile markets have been found to be efficacious in positively influencing F&V intake [36, 37].

**Figure 1:**
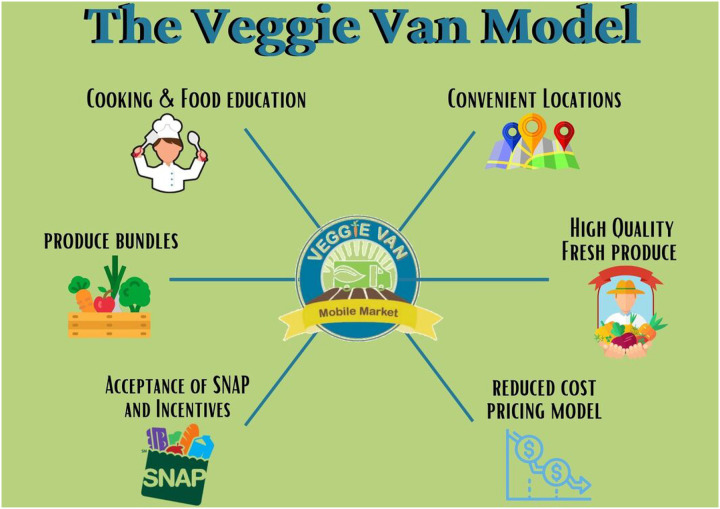
The Veggie Van Model Components

The Veggie Van (VV) model is an evidence-based model intended for mobile markets that has been found efficacious in increasing F&V consumption [37, 39]. Figure 1 depicts the core components of the VV model. The model advises mobile market practitioners to follow six strategies 1.) operate the mobile market on a consistent schedule in intentionally selected underserved, higher-need communities, and partner with local organizations to host the market within those communities, leveraging partners that already serve lower-income populations., 2.) offer a variety of fresh, high-quality F&V, 3.) operate a reduced cost payment model, 4.) accept federal supplemental nutrition assistance program (SNAP) benefits and other available regional incentive programs, 5.) offer regular cooking and nutrition education, and 6.) offer, and incentivize customers to purchase, a bundle of produce (multiple items for a set price) rather than just one or two items separately [37, 39].

Although the VV model has been found to be efficacious in a randomized controlled trial in 12 lower-income communities in North Carolina [37], its effectiveness needs to be demonstrated on a broader scale while the intervention is implemented by community practitioners (i.e., non-research staff) across multiple organizations. However, outcome data on its own is incomplete without an understanding of the implementation of the intervention by those organizations [40–42]. Implementation science underscores the importance of implementing an intervention with fidelity, or how it was intended to be implemented, to ensure that the intervention remains effective in real-world settings [40, 41, 43–45]. A review of over 500 studies evaluating prevention and health promotion programs found that better implementation (e.g., fidelity, dosage, reach) has resulted in mean effects sizes for health outcomes that are two to three times higher in treatment groups compared to control groups [40]. Therefore, the quality of implementation influences outcomes; in the case of mobile markets, poor quality of implementation can translate to a reduced impact on dietary changes (i.e., F&V intake). Understanding implementation of evidence-based practices is especially important in the context of a mobile market, run by a community-based organization, as we recognize that these organizations face numerous challenges to operate and sustain them [32, 33, 46, 47]. In addition, there is a gap in implementation research for evaluating the association between implementation and participant outcomes [40, 42, 43]. We failed to observe a significant association between VV intervention group and our main participant outcomes (F&V intake, food security) [48], This research is crucial for elucidating whether the failure to observe significant differences in main outcomes was due to an ineffective intervention or due to ineffective implementation [40, 42, 43, 49], Therefore, the aim of this paper is to assess fidelity to the VV model among implementers and examine associations between overall and component-specific implementation levels and participant dietary outcomes (F&V intake) and food security, with the goal of identifying the components most strongly associated with these outcomes. Findings will help address gaps in implementation research and advance understanding of the key practices required for mobile markets to support positive dietary change. In addition, identifying the most impactful components will help refine the model’s essential elements and inform improvements to implementation supports (e.g., toolkit, technical assistance).

## METHODS

The Veggie Van study was a hybrid type II effectiveness-implementation randomized controlled trial (RCT) study that evaluated whether the VV model is effective when implemented by multiple mobile market organizations across 33 communities in 5 states [50]. Organizations received funding to start or expand mobile market operations following the VV model; key implementers from each organization also received training and ongoing technical assistance over the 12-month study period. Each organization identified pairs of community sites that would serve as host sites for their mobile market; sites were located in a lower-income and/or low food access community. One site from each pair was randomized to be a market site, operating for at least 12 months and implementing the VV model. The other paired site was assigned to a planning condition (i.e., delayed-intervention control) that entailed a year-long community engagement process with the goal of hosting a market or another food access program after a 12-month planning period. This paper presents the findings from a post-hoc analysis of the Veggie Van RCT effectiveness outcomes, examining how dietary and food security outcomes are associated with implementation outcomes assessed through a concurrent process evaluation.

### Implementation Study Participants

The partner recruitment process is discussed in detail elsewhere. Briefly, nine mobile market organizations were identified through a request-for-partners (RFP) process; additional details regarding recruitment of mobile market organizations through the RFP process are reported elsewhere [51].

### Effectiveness Study Participants

Organizations facilitated recruitment for study participants in partnership with community sites; study participants were lower-income individuals that frequented community sites and were interested in shopping at a future mobile market. Additional details on methods and the results of the effectiveness arm of this study have been reported elsewhere [50, 52]. Briefly, mobile market organizations facilitated recruitment for study participants through a ≈ 2-month community engagement period that preceded the mobile market launch at intervention sites, or initiation of planning activities at control sites. Mobile market organizations worked with stakeholders at community sites, at both intervention and control sites, to identify individuals who expressed that they would be likely to use a mobile market. Interested individuals completed an interest form that was shared with the research team; eligible individuals that indicated interest in the study on their interest form were contacted by the research team for enrollment. If recruitment goals were not met within the initial community engagement period, recruitment continued for up to two months after the mobile market was launched or commencement of planning activities. Eligibility criteria included: at least 18 years old, able to speak English and/or Spanish, be the primary grocery shopper for their household, and live near or otherwise regularly frequent the site. Individuals were ineligible if they were planning to leave the area or stop frequenting the community site within the next year.

### Outcome Measures and Data Collection Procedures

#### Implementation Outcome: Fidelity to the Veggie Van Model

In tandem with implementing the VV model at market sites, mobile market organizations participated in a mixed methods process evaluation [53]. To assess fidelity to the VV model and the dose delivered, quantitative process data was collected through monthly process measures surveys. The process measures surveys were informed by Proctor et al.’s taxonomy of implementation outcomes, which includes multiple domains used to evaluated implementation (e.g., acceptability, adoption, appropriateness, feasibility, fidelity, cost, penetration, and sustainability), with this study focusing specifically on fidelity (adherence to the model) and penetration (reach) [41]. The survey was designed to determine how closely organizations adhered to the VV model (fidelity) through questions that assessed whether core components of the VV model (i.e., bundling, nutrition education; etc.) were being implemented in a given month. Surveys also measured the dose delivered of the intervention through questions that assessed to what degree (i.e., frequency, duration) VV model components were implemented.

Process measures surveys consisted of up to 60 questions and assessed implementation of the VV model at each market site over the course of the 12-month intervention period [53]. The survey was sent to a mobile market representative that was previously designated as being the most familiar with implementation of the VV model through a secure web-based (REDCap) and could be completed in about 15 minutes.. The survey was sent on the first day of each month followed by two reminders throughout the month. Research staff monitored completion of surveys on a regular basis and followed up with representatives from organizations regarding missing or incomplete surveys. Process measures survey data was periodically extracted and cleaned over the duration of the study. This allowed for updates to the survey to be made iteratively based on issues that arose earlier in the study and triangulation of data from our qualitative data (i.e., implementation interviews) and/or clarification of responses directly with representatives from the mobile market organizations.

#### Implementation Outcome: Reach

To assess penetration of the intervention, or reach, organizations were each provided with an iPad and a custom iPad-based Point-of-Sale (POS) software known as Farmers Register © (Perigee Labs, Inc.) to collect individual level purchasing data at market sites. Site-level purchasing data was used to assess the reach of the intervention within each organization’s target community. Mobile market staff received training on Farmers Register © POS software directly from the software developer. Organizations were instructed to utilize the software to process the transactions of all customers, irrespective of if they are study participants, who shopped at participating market sites implementing the VV model. De-identified purchasing data was automatically transmitted to a data portal, Farmers Register Market Reporting (FRMR) portal, maintained by the software developer that could be accessed by the research team. Available data included customers’ loyalty ID number, itemized transaction data including sales amounts and product purchased, and forms of tender used.

### Effectiveness Outcomes: Change in F&V intake and Food Security

The main effectiveness outcome was change in F&V intake (servings/day) between baseline and 12-month follow-up as measured via phone survey using the 2017 Behavioral Risk Factor Surveillance System (BRFSS) Fruit and Vegetable module [54]. Change in average F&V intake was also assessed from 24-hour dietary recall data; the NDSR F&V variable includes all fruits, juices and vegetables consumed. Food security status in the past 12 months was also measured using the USDA 10-item US Adult Food Security Survey [55]. Resulting raw scores were analyzed as a continuous outcome.

### Data Analyses

To aggregate process measures data to assess implementation of the VV model, we developed a fidelity scoring system. Our approach was modeled after past research in the education field that has developed a rubric which awards points based on fidelity to the core tenets of an intervention and creates a resulting score [56]. Table 1 presents the fidelity scoring system details as well as example questions from the process measures survey. This scoring system allows for comparison of implementation over time, within and between organizations. The criteria for the scoring rubric were informed by the research team’s knowledge of how the VV model was implemented in the initial pilot and efficacy studies, but there was no pre-established optimal dosage for each VV model component (e.g., frequency of nutrition education). Therefore, criteria for dosage of the components and the corresponding points awarded were guided by discussion among the PI and research team and informed by experience working with mobile market practitioners and understanding what practices are feasible. In addition to yes/no questions, for some components whose implementation may have varied throughout the month (e.g., a market offered a bundle two weeks out of the month), we also collected frequency data to quantify the dose delivered of those components.

Data from each mobile market site was extracted from the REDCap platform and organized by VV model component, grouping items that correspond to each component. The fidelity rubric was applied to each mobile market site’s data to calculate a monthly fidelity score. Monthly fidelity scores were comprised of sub-scores for each VV model component that were added to create an overall site-level monthly fidelity score. Annual fidelity scores were comprised of sub-scores for two of the VV model components (convenient location, pricing model). All monthly scores for a site were averaged across operating months (up to 12 months) and added to the annual site score to create a total site-level fidelity score. Fidelity scores were calculated in Excel; redundancy measures including calculating scores manually as well as with formulas were put in place to ensure the validity of calculated scores.

Farmers Register © purchasing data was aggregated at the site level in Excel. Variables include total number of transactions, total sales, and the average transaction amount over the 12-month study period. One organization did not use the provided POS software; therefore, sales data was only available for 17 mobile market sites. For the main effectiveness outcomes, F&V intake and food security, change scores were calculated to assess the difference between 12-month follow-up and baseline. The overall mean change for each outcome was calculated at the site level for all market sites. Extreme F&V reporters, defined as participants who had a change (increase or decrease) greater than 10 servings of F&V per day, were removed from analyses before calculating the site-level mean change. This threshold was informed by the past VV efficacy study[37] and further justified by generating histograms to identify outliers in the data distribution for the current study. Fidelity scores, sales data, and mean change scores (F&V intake and food security) were integrated into a single site-level dataset for analysis.

All analyses were completed using SAS 9.4. Descriptive statistics were calculated at the site and organization levels, such as average monthly, annual, and total scores for VV model components across organizations. We conducted correlational analyses between fidelity scores and mean changes in F&V consumption and food security. We then conducted generalized linear model (GLM) regression to further explore the association between implementation outcomes and mean changes in dietary outcomes. We conducted GLM regression to evaluate the association between fidelity scores and site-level sales outcomes. We also conducted sensitivity analyses using the participant-level dataset from the effectiveness analyses to examine the same association by incorporating fidelity scores as variables assigned to all study participants that were randomized to market sites. Lastly, we conducted a logistic regression to understand the relationship between implementation outcomes and the likelihood of study participants at market sites becoming a shopper, or VV user. For the sensitivity analysis using a logistic regression model, the total duration of mobile market site operation was categorized into a dichotomous variable (long versus short/medium seasons).

## RESULTS

### Summary Statistics for Implementation, Dietary, and Sales Outcomes

Table 2 presents summary statistics on for implementation, dietary, and mobile market sales outcomes. Seventeen market sites across nine organizations implemented the VV model. The mean total organizational-level fidelity score was 39.77 out of 55 maximum possible points (range: 30–49.2). The mean mobile market site level fidelity score was 40.6 out of 55 points (range: 30–50.8). The VV model components annual pricing model and monthly convenient location had the highest scores in comparison to other components; in contrast, nutrition education and bundling had lower mean scores across organizations.

Fruit and vegetable intake changed modestly overall, with a small average increase based on 24-hour dietary recall and a small decrease based on self-report survey measures; variability was higher for the recall-based measure. Mobile market sales outcomes showed substantial variability. Total transaction dollars and total sales amounts varied widely across the study period, while the number of transactions demonstrated more moderate variability.

### Associations Between Implementation and Effectiveness Outcomes

Table 3 shows results from correlational analyses between mean change in dietary outcomes and food security scores in relation to site-level implementation outcomes. There was a positive (r = 0.81) and statistically significant correlation (p < .0001) between mean change in F&V intake, measured through survey, and the nutrition education 12-month average score. The overall site fidelity score at 12 months was also significantly (p = 0.02) and positively correlated (r = 0.56) with mean change in F&V consumption measured through survey. In addition, the average monthly fidelity score was positively (r = 0.70) correlated with mean change in F&V consumption (p = 0.002). These significant associations remained in linear regression models (see Table 4). There were no significant associations between implementation and changes in food security in our correlation or regression analyses.

In sensitivity analyses, we evaluated the same associations but using the larger VV study participant dataset with implementation variables associated with each participant based on the market site they were recruited from. In these linear regression analyses, the only association that was significant was between nutrition education 12-month average score and mean change in F&V intake measured through survey (0.09; p = 0.03). Study participants were considered VV “users” if they indicated on their 12-month follow-up survey that they had shopped at the mobile market at least once during the intervention period. A logistic regression model evaluating the association between implementation outcomes and participants’ user status indicates that operating a market for 10 or more months out of the year, compared to markets that operated for a shorter length of time, resulted in 76% greater odds of participants frequenting the mobile market at least once (p = 0.04). Furthermore, the odds of becoming a VV user increased as the monthly convenient location score increases, which captures whether a market remains in the same location and operates regularly within a certain month (OR: 1.63; p = 0.0047).

### Associations Between Implementation Outcomes and Market Sales Outcomes

Table 5 presents the results from regression analyses between implementation outcomes and market sales outcomes (i.e., reach). The average monthly fidelity score was significantly associated with total market sales over the intervention period (p = 0.03). With every one-point increase in monthly fidelity score, there is a resulting increase of about $584 in total sales over the 12-month intervention period. Total site-level fidelity score (monthly and annual scores combined) at 1 year was also significantly associated with total sales amount (p = 0.01) as well as the total number of transactions (p = 0.02). Every one-point increase in the total fidelity score results in about $560 increase in total sales and an increase of 26 total transactions. There were no significant associations between implementation outcomes and average transaction amount.

## Discussion

This process evaluation of VV implementation by mobile market organizations advances our understanding of how fidelity to specific model components is associated with dietary change and intervention reach. The results indicate that high fidelity to the VV model component nutrition education is associated with a greater impact on self-reported F&V intake over time. If a site attained the highest possible score (10 out of 10) for implementing the component of nutrition education, this would amount to an increase in .47 servings of F&V. Further, site-level fidelity to the VV model as a whole is also associated with an increase in F&V consumption at follow-up. If a site attained an average monthly fidelity score of 40, this would amount to an increase in .32 servings of F&V. Better adherence to the VV strategy of ensuring convenient location and consistent operations, which captures how present the mobile market is on a monthly basis, is correlated with a higher odds of study participants becoming shoppers. In addition, compared to mobile market sites that operated for less than 10 months, sites that operated for greater than or equal to 10 months resulted in higher odds of study participants becoming shoppers. Lastly, our analyses indicate that site-level fidelity to the VV model as a whole was associated with increased market sales and total transactions.

Although overall site-level implementation fidelity was associated with F&V intake in primary analyses, this relationship did not remain in sensitivity analyses using participant-level effectiveness data. This may be due, in part, to limited variability in several core components of the model (e.g., pricing, procurement, and location), which reduces the ability to explain differences in outcomes. However, their lack of correlation should not be seen as a reason to discount their importance. In contrast, nutrition education—one of the more variable components—remained significantly associated with changes in intake, suggesting it may be a key driver of dietary behavior change. Bundling, which also showed greater variability and a marginally significant p-value, may similarly warrant further exploration. Although site-level fidelity to the model was associated with increased market sales and total transactions, the individual model components as well as the total months operated were not associated with sales outcomes. Perhaps the site-level fidelity scores, both at the annual and monthly level, are indicative of some other organizational factors such as systematic planning or structure that leads to increased customers and transactions.

Findings from this research help explain why we may have failed to observe significant association between intervention group and dietary outcomes in our main outcome analyses [48]. In our main outcome analysis, food security was significantly associated with intervention groups, but only among mobile market shoppers at sites that launched after peak COVID-19 (i.e., post-COVID sites) closures. However, we failed to observe any association between implementation outcomes and changes in food security scores in this study. Given that our sample size is already limited by the small number of mobile market sites (n = 17), we did not conduct additional analyses within the post-COVID subset of sites and study participants. Collectively, these findings suggest that operating a mobile market may be sufficient to influence food security among shoppers. However, greater fidelity to specific VV model components, particularly nutrition education and season length, will influence F&V intake, and enhance reach, respectively.

Our findings align with our qualitative implementation research with mobile market organizations, including those that participated in the VV effectiveness study. Barriers to implementing nutrition education by a mobile market organization have included lack of capacity (e.g., staff, expertise) as well as poor weather, and pandemic-related closures resulting in a lack of alternate space to operate indoors. Regulatory barriers, particularly during the COVID-19 pandemic, have hindered organizations’ ability to secure the necessary licensure to conduct food demonstrations. In addition, the lack of resources (e.g., washing station) and increase in costs (e.g., staff time) are barriers to implementing food and nutrition education. However, the VV model has been perceived by mobile market organizations as intentional and consistent with the integration of nutrition education strengthening the collective impact of the model components.

For the VV model component of convenient location, season extension presents challenges with operating the market in inclement weather which greatly impacts staff comfort and customer traffic. Extending the season year-round can also increase operational costs and prove challenging to procure high quality produce in certain climates. Furthermore, a lack of regional or local options for produce procurement during off-peak months may be an intractable problem for organizations that are inflexible in their mission (e.g., local produce only) and unwilling to procure non-local produce. Although, organizations have acknowledged that there are advantages to extending the season such as demonstrating commitment to a community and fulfilling a need among customers to continue to provide access during the winter.

This research has several limitations. Given that the organizations participating in the VV study received ongoing technical assistance and funding from the research team, they may be better equipped or supported to implement the VV model. Future studies can evaluate implementation among a more diverse sample of organizations that are not actively receiving technical assistance and funding. In addition, partway through the VV study, we identified several areas of the process measures survey that were interpreted differently between organizations. We determined that there is varying lexicon within the mobile market space that needed to be considered in developing our instruments. For example, the original process measure survey asked about pricing model and one of the responses was intended to represent a free food distribution model but was misunderstood by some as a “pay-what-you-can” pricing model. The process measures survey was also developed pre-pandemic and was not designed to account for pandemic adaptations such as virtual markets and nutrition education. The process measures survey was amended to enhance comprehension, account for possible adaptations, as well as more precisely capture fidelity to the VV model components through more detailed questions. However, despite these improvements, data collected earlier in the study likely overestimated implementation (i.e., fidelity) of the model. Future studies should pilot test process evaluation instruments to address issues prior to implementation. There was also missing process measures data; however, missingness was minimal as sites completed an average of 11 months of process measures survey data during the main study period. We were also able to triangulate missing data with qualitative implementation interview data when available.

The strength and generalizability of our findings are limited by the small number of participating mobile market sites (n = 17). To address this, we conducted sensitivity analyses by merging site-level fidelity scores into the participant-level effectiveness dataset (n = 426), allowing examination of associations while accounting for individual participant variation. Primary analyses suggested multiple associations between implementation and dietary outcomes; however, only one association remained significant in sensitivity analyses. These results highlight a promising relationship between implementation and dietary outcomes and indicate that future studies with a larger number of sites are needed to explore other potential associations. Additionally, due to inconsistent or underutilization of the provided POS software over the study period, purchasing data was likely underreported. Despite these limitations, we still observed significant associations between implementation and sales outcomes, and a larger sample of sites would likely further strengthen these relationships. Lastly, most organizations failed to collect demographic information when processing transactions on the POS software, which limits our ability to determine whether the intervention reached its intended audience of individuals with limited healthy food access.

## Conclusions

Our findings can inform future implementation efforts to enhance the impact of the VV model on F&V intake, food security, and market sales. Support and resources for organizations implementing the model, such as tailored technical assistance, are needed to support the implementation of the VV model. In particular, support for implementing the components nutrition education and convenient location (i.e., extending the market season) may enhance the impact of the model on dietary outcomes. Our findings from implementation interviews with study organizations highlighted several areas where targeted support from technical assistance (TA) providers and other stakeholders could be particularly impactful. Reliable funding has consistently been noted by organizations as facilitating operations in general, and implementation of the VV model. TA providers and stakeholders can provide guidance on navigating regulatory requirements related to food demonstrations and can facilitate partnerships with organizations that can support nutrition and food education. Mobile market organizations would also benefit from assistance with strategies to help extend the season, such as accessing indoor market space, guidance on weatherizing mobile markets, and support for sourcing year-round produce or developing season-extension infrastructure such as greenhouses. Finally, support for implementing adaptations to the VV model, including virtual nutrition education and alternative approaches to offering produce bundles, is needed.

It is important to note that, although we did not observe significant associations for some VV model components—such as produce bundling and high-quality produce procurement—this does not necessarily indicate that these components are ineffective. The absence of observed associations may reflect the small sample size and/or limited variability in how these components were implemented. It is also important to acknowledge that, although better implementation was associated with increased total sales, this does not necessarily guarantee enhanced sustainability of the intervention. Higher sales may reflect greater reach or customer engagement in the short term, but sustaining operations over time likely depends on consistent resources, staffing, and supportive infrastructure. Future research can explore the relationship between VV model implementation and financial sustainability, particularly implementation of the reduced cost payment model component.

We did not observe a significant association between implementation outcomes and food security. The VV model component of convenient location, reflecting regular (i.e., weekly) operation of the mobile market, had a high mean (9.43 out of 10) and a narrow range (6.25–10), indicating that most sites consistently provided access to the market. This limited variability, combined with the small number of sites, likely reduced our ability to detect associations. Consistent with this, in the effectiveness arm of the VV study, significant improvements in food security were observed only among participants who shopped at the market and at sites that launched after peak COVID-related restrictions. Together, these findings suggest that the VV model can improve food security through program engagement alone, greater fidelity to the model may be necessary to influence dietary behaviors.

Lastly, our approach for aggregating, scoring, and analyzing implementation data alongside effectiveness outcomes can serve as a model for similar process evaluations. However, refinements to our scoring rubric may be needed to ensure that implementation of VV model components are accurately captured, sufficiently differentiated, and appropriately weighted according to their importance within the overall VV model. Future refinements could strengthen the ability to detect meaningful associations between implementation fidelity and effectiveness outcomes. They could also improve the utility of this approach as a model for developing and applying comparable fidelity scoring rubrics in similar intervention settings.

## Figures and Tables

**Table 1:** Veggie Van Model Fidelity Scoring Criteria

VV Model Component	Monthly Fidelity Score Criteria	Annual Fidelity Score Criteria	Example Questions from Process Measures Survey
**1.) Convenient Location**	**Did the organization remain at the host site the entire time or relocate to a nearby location serving the same community?** *[Yes: 5 pts; No: 0 pts]*	**How long was the operating season?** *[10–12 months: 5 pts; 8–9 months: 4 pts; 6–7 months: 3 pts; less than 6 months: 0 pts]*	*“How many times was a mobile market program held at [site name] in the last calendar month?”* *“When the market was held last month, was it always located directly at [site name]?*
	**Did the organization operate at least 3 times per month in operating months?** *[Yes: 5 pts; No: 0 pts]*		
**2.) High Quality Produce Procurement**	**Procuring high quality produce?** *[At least some local AND no rescued food: 5 pts; At least some local AND some rescued food: 3pts; No local AND/OR exclusively rescued food: 0 pts]*		*Where do you most often get produce for this [site name] last month?* *“About what percentage of the produce offered at the market last month was locally sourced?”*
**3.) and 4.) Reduced Cost Pricing Model**	**Implementing a reduced cost pay model?** *[Yes: 5 pts; No: 0 pts]*	**Participate in the SNAP Program?** *[Yes: 5 pts; No: 0 pts]*	*“What type of pricing model are you offering at this market site?”* *“What types of incentives are accepted at this market site?”*
		**Participate in at least one incentive program over the course of the year?** *[Yes: 5 pts; No: 0 pts]*	
**5.) Nutrition Education**	**Implemented some form of food lesson and/or demonstration?** *[Yes: 5 pts; No: 0 pts]*		*“Was a food or nutrition lesson/activity offered at the market at [site name] at least once last month?”* *“How often was a nutrition lesson offered at the market?”*
	**Frequency of implementing food lessons and/or demonstration?** *[At least bi-weekly (2 or more times): 5 pts; monthly (1 time per month): 3 pts; Never: 0 pts]*		
**6.) Bundling**	**Implemented a bundle with an incentive?** *[Yes: 5 pts; Yes, but without an incentive: 3 pts; No: 0 pts]*		*“Was a bundle offered at the market last month?”* *“How many days did the market offer a bundle last month?”*
	**Frequency of implementing a bundle?** *[Weekly (3 or more times): 5 pts; bi-weekly: 3 pts; less than 2 times per month: 0 pts]*		
**Maximum Possible Score**	40	15	
	**Average of Site’s Monthly Fidelity Scores + Annual Fidelity Score =Total Fidelity Score [Maximum possible score: 55]**			

**Table 2:** Summary Statistics for Implementation, Dietary, and Sales Outcomes

Implementation Outcomes (Independent Variables)				
	Mean Score (SD)	Minimum	Maximum	n [Number of market sites]
***Monthly Fidelity scores***				
**Convenient Location** **Maximum Score Possible: 10**	9.43 (0.9)	6.25	10	17
**Nutrition Education** **Maximum Score Possible: 10**	3.39 (2.81)	0	8.2	17
**Bundling** **Maximum Score Possible: 10**	4.9 (3.53)	0	10	17
**Pricing Model** **Maximum Score Possible: 5**	4.94 (0.24)	4	5	17
**Produce Procurement** **Maximum Score Possible: 5**	4.85 (0.37)	3.5	5	17
**Average Total Monthly Score** **Maximum Score Possible: 40**	27.48 (5.77)	20	38	17
**Annual Fidelity scores**				
**Convenient Location** **Maximum Score Possible: 5**	4 (1.88)	0	5	17
**Pricing Model** **Maximum Score Possible: 10**	9.12 (1.91)	5	10	17
**Total Fidelity scores**				
**Mobile Market Site Level Fidelity score** **Maximum Score Possible: 55**	40.6 (6.53)	30	50.8	17
**Total Organization-Level Fidelity score** **Maximum Score Possible: 55**	39.77 (6.19)	30	49.2	9
**Dietary Outcomes (Dependent Variables)**				
***Monthly Fidelity scores***				
**Mean Change in total Fruit and Vegetable Intake (servings) Measured through 24-hr Dietary Recall**	0.20 (1.72)	−2.72	5.34	17
**Mean Change in total Fruit and Vegetable Intake (servings) Measured through Self-report Survey**	−0.26 (0.38)	−0.87	0.61	17
**Mean Change in Food Security Score**	−0.46 (0.61)	−2	0.56	17
**Mobile Market Sales Outcomes (Dependent Variables)**				
**Total Transaction Amount (Dollars)**	5081.12 (5165.56)	114.95	18771	17
**Total Sales Amount (Dollars)**	358.86 (258.96)	22	832	17
**Total Transactions**	12.04 (5.45)	4.6	23.58	17

**Table 3:** Correlational Analyses Evaluating Associations Between Implementation and Effectiveness Outcomes

Implementation Outcomes	Effectiveness Outcomes						
	Mean Change in total Fruit and Vegetable Intake (24-hr Dietary Recall)	P-value	Mean Change in total Fruit and Vegetable Intake (Self-report Survey)	P-value	n	Mean Change in Food Security Score	P-value
**Convenient Location 12-Month Average**	−0.02	0.94	0.20	0.44	17	−0.25	0.34
**Nutrition Education 12-month Average**	0.47	0.05	0.81	< .0001	17	−0.37	0.14
**Bundling 12-month Average**	0.27	0.29	0.46	0.06	17	−0.32	0.21
**Pricing Model 12-month Average**	−0.17	0.51	0.02	0.94	17	−0.15	0.56
**Produce Procurement 12-month Average**	−0.14	0.60	0.06	0.81	17	−0.25	0.34
**Average Monthly Fidelity score**	0.36	0.15	0.70	0.002	17	−0.44	0.08
**Annual Convenient Location Score**	0.18	0.49	−0.15	0.56	17	0.30	0.24
**Total Months Operated**	0.08	0.77	−0.16	0.55	17	0.13	0.62
**Pricing Model Annual Score**	0.001	1.00	−0.08	0.77	17	0.000	0.99
**Total Mobile Market Site Fidelity score at 1 year**	0.37	0.14	0.56	0.02	17	−0.30	0.24

Pearson’s correlation coeffi cient (r)

**Table 4:** Regression Analyses Evaluating Associations Between Implementation and Effectiveness Outcomes

Implementation Outcomes	Effectiveness Outcomes						
	Mean Change in total Fruit and Vegetable Intake Measured through 24-hr Dietary Recall (SE)	P-Value	Mean Change in total Fruit and Vegetable Intake Measured through Self-report Survey (SE)	P-Value	Mean Change in Food Security Score (SE)	P-Value	n
**Convenient Location 12-Month Average**	−0.04 (0.49)	0.94	0.08 (0.11)	0.44	−0.17 (0.17)	0.34	17
**Nutrition Education 12-month Average**	0.29 (0.14)	0.05	0.11 (0.02)	< .0001	−0.08 (0.05)	0.14	17
**Bundling 12-month Average**	0.13 (0.12)	0.29	0.05 (0.02)	0.06	−0.06 (0.04)	0.21	17
**Pricing Model 12-month Average**	−1.26 (1.85)	0.51	0.03 (0.42)	0.94	−0.4 (0.66)	0.56	17
**Produce Procurement 12-month Average**	−0.65 (1.2)	0.60	0.06 (0.27)	0.81	−0.41 (0.41)	0.34	17
**Average Monthly Fidelity score**	0.11 (0.07)	0.15	0.05 (0.01)	0.0016	−0.05 (0.02)	0.08	17
**Annual Convenient Location Score**	0.17 (0.23)	0.49	−0.03 (0.05)	0.56	0.1 (0.08)	0.24	17
**Total Months Operated**	0.05 (0.18)	0.77	−0.02 (0.04)	0.55	0.03 (0.06)	0.62	17
**Pricing Model Annual Score**	0.0009 (0.23)	0.99	−0.02 (0.05)	0.77	−0.0008 (0.08)	0.99	17
**Total Mobile Market Site Fidelity score at 1 year**	0.1 (0.06)	0.14	0.03 (0.01)	0.02	−0.03 (0.02)	0.24	17

**Table 5:** Regression Analyses Evaluating Associations Between Implementation and Market Sales Outcomes

Implementation Outcomes	Market Sales Outcomes						
	Average Transaction Amount (SE)	P-Value	Total Sales Amount (SE)	P-Value	Total Transactions (SE)	P-Value	n
**Convenient Location 12-Month Average**	1.28 (1.62)	0.45	1924.09 (1478.54)	0.22	93.51 (74.44)	0.23	14
**Nutrition Education 12-month Average**	0.32 (0.57)	0.59	810.31 (496.83)	0.13	43.24 (24.54)	0.10	14
**Bundling 12-month Average**	0.54 (0.51)	0.31	779.97 (451.91)	0.11	35.03 (23.2)	0.16	14
**Pricing Model 12-month Average**	5.16 (5.92)	0.40	2497.52 (5745)	0.67	−23.85 (290.18)	0.94	14
**Produce Procurement 12-month Average**	4.97 (3.71)	0.20	2476.19 (3699.83)	0.52	−26.32 (188.76)	0.89	14
**Average Monthly Fidelity score**	0.36 (0.29)	0.24	583.74 (241.53)	0.03	26.99 (12.54)	0.05	14
**Annual Convenient Location Score**	0.21 (0.91)	0.82	1025.48 (811.56)	0.23	76.12 (37.32)	0.06	14
**Total Months Operated**	−0.24 (0.64)	0.72	338.88 (605.09)	0.59	33.77 (29.14)	0.27	14
**Pricing Model Annual Score**	0.96 (0.71)	0.20	700.18 (698.15)	0.34	15.41 (36.16)	0.68	14
**Total Mobile Market Site Fidelity score at 1 year**	0.37 (0.23)	0.14	559.7 (182.34)	0.01	26.25 (9.58)	0.02	14

## Data Availability

The datasets used and/or analyzed during the current study are available from the corresponding author on reasonable request.

## Abbreviations

BRFSS: Behavioral Risk Factor Surveillance Survey
COVID-19: Cornonavirus disease 2019
F&V: Fruit and Vegetable
FRMR: Farmers Register Market Reporting
GLM: generalized linear model
NDSR: Nutrition Data Systems for Research
OR: Odds Ratio
PCC: Pearson correlation coefficient
POS: Point-of-Sale
RCT: Randomized Controlled Trial
RFP: Request-for-partners
SNAP: Supplemental Nutrition Assistance Program
TA: Technical assistance
US: United States
USDA: United States Department of Agriculture
VV: Veggie Van
